# Solvent Effects on C–H Abstraction by Hydroperoxyl Radicals: Implication for Antioxidant Strategies

**DOI:** 10.1021/acs.joc.5c01140

**Published:** 2025-09-15

**Authors:** Andrea Baschieri, Zongxin Jin, Greta Tödtmann, Gino A. DiLabio, Riccardo Amorati

**Affiliations:** † 201838 Institute for Organic Synthesis and Photoreactivity (ISOF), National Research Council of Italy (CNR), Via P. Gobetti 101, I-40129 Bologna, Italy; ‡ Department of Chemistry “G. Ciamician”, University of Bologna, Via Gobetti 83, 40129 Bologna, Italy; § Department of Chemistry, 97950 The University of British Columbia, 3247 University Way, Kelowna, British Columbia V1 V 1 V7, Canada

## Abstract

Kinetic solvent effects (KSE) on hydrogen atom transfer (HAT) reactions play a pivotal role in processes such as photoredox catalysis, electrochemical synthesis, and antioxidant defense. While general principles of KSE are well established, the influence of solvent-radical interactions on the reactivity of the hydroperoxyl radical (HOO^•^) remains largely uncharacterized. Here, we examine the effects of noncovalent interactions and acid–base equilibria on HOO^•^ reactivity, using the autoxidation of 1,4-cyclohexadiene (CHD) as convenient HOO^•^ source in chlorobenzene (PhCl) or acetonitrile solutions containing cosolvents (S) with varying hydrogen bond acceptor basicities (β_2_^H^). Equilibrium (*K*_S_) and CHD + HOO^•^ (*k*_p_^S^) rate constants in PhCl were determined for cosolvents including MeOH, MeCN, DMSO, pyridine, and DABCO. As β_2_^H^ increased from 0.41 (MeOH) to ∼0.70 (DABCO), *K*_S_ increased from 50 to 3 × 10^6^ M^–1^, while *k*_p_^S^ decreased from 90 to 0.1 M^–1^ s^–1^. MeCN (β_2_^H^ = 0.44) gave *K* ^S^ = 70 M^–1^ and *k*_p_^S^ = 130 M^–1^ s^–1^. For DMSO (β_2_^H^ = 0.78) and pyridine (β_2_^H^ = 0.62) *K*_S_ values were 2.0 × 10^3^ and 3 × 10^5^ M^–1^, respectively, with corresponding *k*_p_^*S*^ values of 20 and 5 M^–1^ s^–1^. The observed *K*_S_ values show a qualitative correlation with the solvent β_2_^H^ values of the solvents. Moreover, the calculated α_2_^H^ values for HOO^•^ in nonbasic cosolvents (MeOH, MeCN, DMSO) cluster around 0.87 ± 0.07, consistent with prior estimates. Experiments in MeCN solution suggest HOO^•^ deprotonation with alkylamines, and the p*K*_a_ of HOO^•^ is estimated as 18–19. These findings provide mechanistic insight into HOO^•^ reactivity in complex media and suggest new strategies for modulating oxidative radical chemistry in both synthetic and biological contexts.

## Graphical abstract

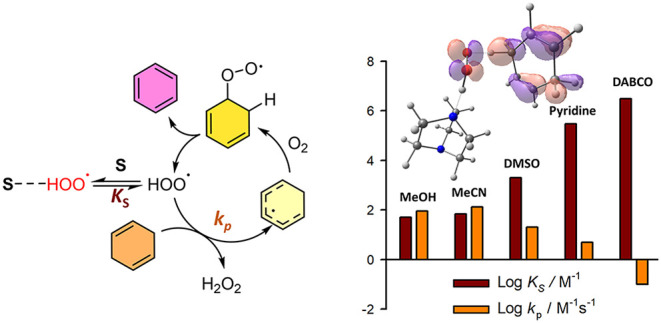

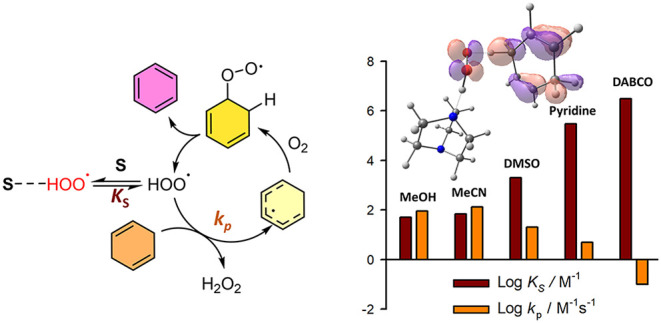

## Introduction

Kinetic solvent effects (KSE) in hydrogen atom transfer (HAT) reactions are of great interest because they occur in a variety of processes including photoredox catalysis,^1–3^ electrochemical synthesis,^4^ and lipid peroxidation inhibition by antioxidants.^5–7^ In the late 1990s, Ingold and co-workers established that H atoms of polar X–H groups (i.e. X = O, N) donating a H-bond to solvent molecules cannot be abstracted by “any” radical (Y^•^), which includes both practically relevant species such as alkyl, alkoxyl, and peroxyl radicals, as well as model compounds like diphenylpicrylhydrazyl (DPPH^•^) (Figure 1A).^8^ Since the Y^•^ radicals examined in these seminal works could only form weak interactions with solvents, the KSE could be predicted using descriptors of the H-bond donating ability of the X–H group and the H-bond accepting ability of the solvent (i.e., by the Abraham α_2_^H^ and β_2_^H^ parameters, respectively).^9^ However, these initial findings inspired additional research into refining or identifying exceptions to this rule. For example, the sequential proton loss–electron transfer mechanism (SPLET) causes large deviations from “conventional” KSE in alcoholic solvents in the case of DPPH^•^.^10,11^ Other deviations are the interactions between the solvent and groups that can influence the reactivity of X–H;^12^ in this case, KSEs are observed in HAT from C–H bonds (i.e., from nonpolar X–H groups), such as in ethers and amines.^13^ Examples of KSE resulting from the interaction of abstracting radicals with the solvent are scarce. The KSE on HAT from C–H bonds by alkoxyl (Y^•^ = RO^•^) and alkylperoxyl (Y^•^ = ROO^•^) radicals is usually minimal, as expected from their poor H-bond accepting or donating ability.^14,15^ Hydroxyl (HO^•^) and hydroperoxyl radicals (HOO^•^) are possible exceptions since they have highly polarized O–H groups, which make these radicals able to interact with solvents, potentially modifying their reactivity. In 2010, Tanko and co-workers reported that, in H_2_O, HO^•^ radicals have enhanced reactivity compared to MeCN.^16^

**Figure 1:**
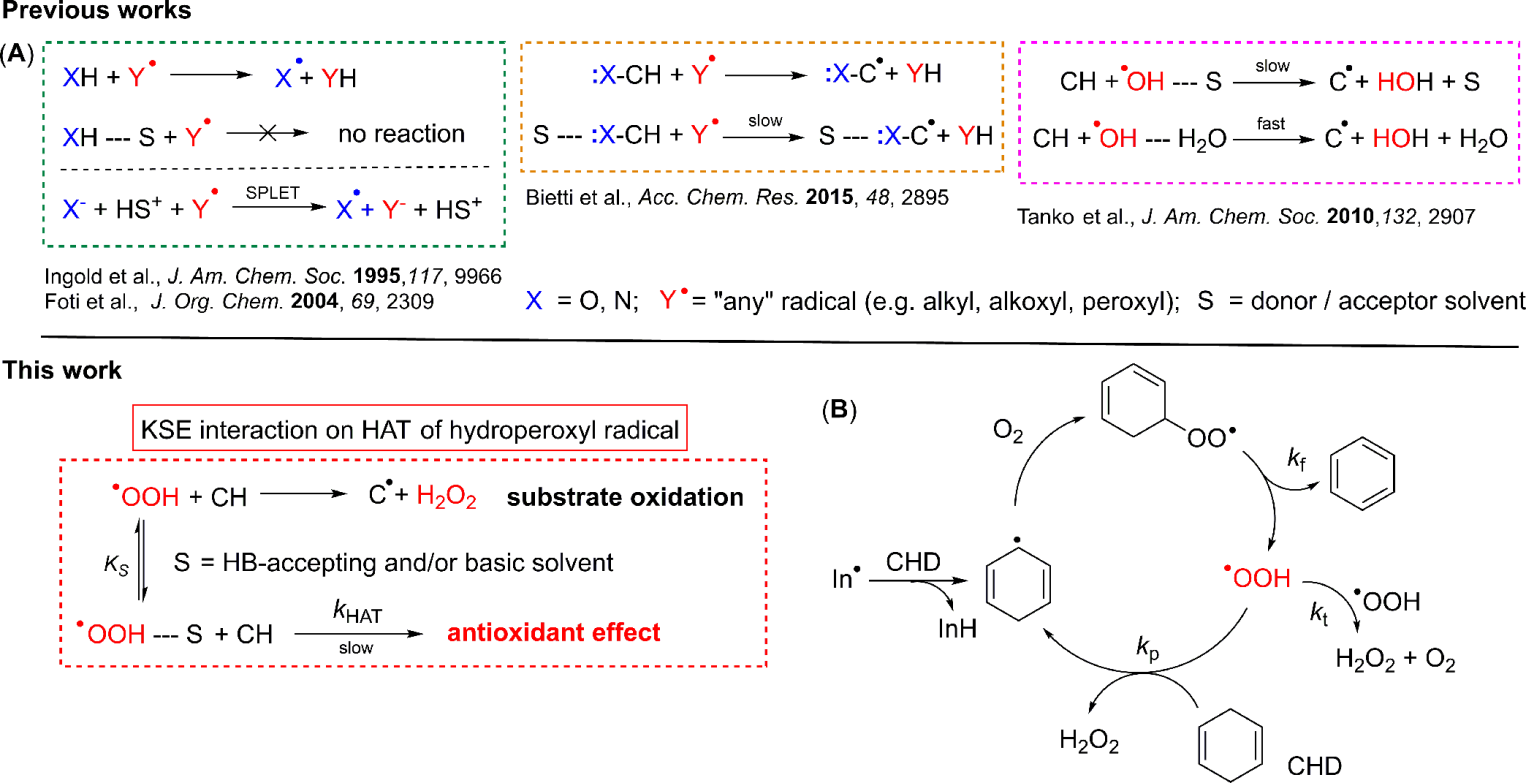
(A) Literature-reported examples of kinetic solvent effects (KSE) in hydrogen atom transfer (HAT) reactions compared to our work; (B) mechanism of CHD autoxidation leading to HOO^•^ radicals. Figure 1A (top) has been adapted from ref 11, Copyright [2007] American Chemical Society and from ref 16, Copyright [2010] American Chemical Society.

Although it is known that HOO^•^ can donate H-bonds,^17–19^ there is limited understanding of kinetic solvent effects (KSE) on the radical in hydrogen atom transfer (HAT) reactions.^20,21^ The HOO^•^ radical and superoxide, its deprotonated form O_2_^•–^ (p*K*_a_ = 4.5),^22^ are reactive oxygen species that play important roles in biochemical processes such as the function of the immune system, signal transduction, oxidative stress and ferroptosis,^23–26^ or have many implications in the syntheses of organic compounds.^27^ The oxidizing power of O_2_^•–^ is low compared to other oxygen-centered radicals (alkylperoxyl, ROO^•^, or alkoxyl, RO^•^),^22^ and in most cases, it behaves as a one-electron donating species, although with strong reductants (like ascorbate) it may act as oxidizer.^28^ Instead, the HOO^•^ radical exhibits both reducing and oxidizing characteristics.^20,22,29^

The goal of this study is to identify the role played by noncovalent interactions on the reactivity of HOO^•^ radical through a detailed investigation of the kinetics of HAT from C–H bonds in the presence of basic and/or hydrogen-bond-accepting cosolvents (S) and demonstrate the practical implications to the development of new antioxidant strategies. We show that KSE on HOO^•^ radicals can be conveniently studied in solution by measuring the rate of autoxidation of 1,4-cyclohexadiene (CHD); the fragmentation of the alkylperoxyl radical of CHD, formed by reaction of the CHD alkyl radical with O_2_, quantitatively affords HOO^•^ (chain-carrying radical)^8^ and benzene (*k*_f_ = 4 × 10^4^ s^–1^)^30^ due to the gain of aromatic stabilization (Figure 1B). The stepwise addition of a cosolvent to CHD autoxidation provides critical insights regarding the magnitude of the reactivity changes due to S···HOO^•^ interactions and the concentration of S at which it occurs.

It will be demonstrated that basic cosolvents provide an unexpectedly strong binding of HOO^•^ radicals, resulting in a large reduction in the H atom abstracting ability of HOO^•^ while having little effect on HOO^•^ dismutation. Consequently, some basic species (including solids such as alumina) exhibit antioxidant activity when mixed ROO^•^/HOO^•^ radicals are the chain-carrying species in an autoxidation process. Some relevant examples of how to exploit this novel property are provided.

## Results and Discussion

### Kinetic Studies of CHD Autoxidation

The reactivity of HOO^•^ radicals was investigated by measuring the rate of autoxidation of a fixed amount of CHD at 30 °C, by using 2,2′azobis­(2-methylpropionitrile) (AIBN) as a source of a constant and reproducible flux of initiating radicals In^•^, see Figure 1B. The rate of O_2_ consumption during CHD autoxidation follows the classical rate law of radical chains,^20,31^ where *k*_p_ and *k*_t_ represent the propagation and termination rate constants, respectively, and *R*_i_ is the initiation rate due to AIBN decomposition (eq 1). The study of the autoxidation kinetics in solution of the natural 1,4-cyclohexadiene γ-terpinene showed that the autoxidation rate is not dependent on O_2_ concentration and that the only propagating radical is HOO^•^, indicating that *k*_f_ is fast enough to ensure that all alkylperoxyl radicals fragment to HOO^•^.^32^

1 $$-\frac{d\left[O_{2}\right]}{dt}=\frac{k_{p}}{\sqrt{{2k}_{t}}}\left[\mathrm{CHD}\right]\sqrt{R_{i}}$$

O_2_ consumption rates (−d­[O_2_]/d*t*) were measured by a high-sensitivity gas uptake recording apparatus.^21,33–35^ The addition of variable amounts of cosolvents had a marked kinetic solvent effect (KSE) on the O_2_ consumption rate (Table S1a and Figures S1–S14). As [CHD] and [AIBN] were kept constant, and small solvent additions do not modify AIBN decomposition and thus *R*_i_ (see Figure S15), the variations of d­[O_2_]/d*t* could be explained by a change of the oxidizability term, $k_{p}/\sqrt{{2k}_{t}}$ .

### Low Polarity Medium

CHD autoxidation was measured in chlorobenzene (PhCl), as a function of the amount of added nonbasic/basic and/or hydrogen-bond accepting cosolvents. Nonbasic cosolvents used in the study included acetonitrile (MeCN) and dimethyl sulfoxide (DMSO), both of which are H-bond acceptors and do not undergo oxidation under the experimental conditions.^20,31,36^ Additionally, methanol (MeOH), which could interact with HOO^•^ also by H-bond donation and is potentially oxidizable at the α-CH_3_ position, was employed as a cosolvent. Triethylamine (TEA) and 1,4-diazabicyclo[2.2.2]­octane (DABCO) were used to investigate the role of base oxidation. Finally, pyridine (Py) and 2,2,6,6-tetramethylpiperidine (TMP) were chosen as strong nitrogen bases that can form hydrogen bonds with HOO^•^ but not be susceptible to hydrogen atom abstraction themselves. In fact, it is well-known that amines undergo relatively fast H atom abstraction from their α-CH_2_ groups by certain radicals,^37^ including ROO^•^.^38^ In the case of MeCN and DMSO (see Figure 2, black and blue dots), which are solvents capable of only accepting H-bonds, a decrease of the oxidation rate of CHD was observed at low cosolvent concentrations. This can be interpreted as a larger reduction of *k*_p_ relative to $\sqrt{{2k}_{t}}$ in the oxidizability term of eq 1. Interestingly, in the case of DMSO, the O_2_ consumption rate increased at [DMSO] > 10 mM. Considering that DMSO has low reactivity toward ROO^•^ or HOO^•^, we exclude the possibility that this observation is due to DMSO autoxidation.^36^ Instead, this result can be explained by considering that at concentrations larger than 10 mM, DMSO reduces $\sqrt{{2k}_{t}}$ more than *k*_p_. Moreover, in neat MeCN or DMSO, the rate of autoxidation of CHD is faster than in PhCl,^20,21^ indicating that at high cosolvent concentrations, there is a larger decrease in $\sqrt{{2k}_{t}}$ compared to *k*_p_.

**Figure 2:**
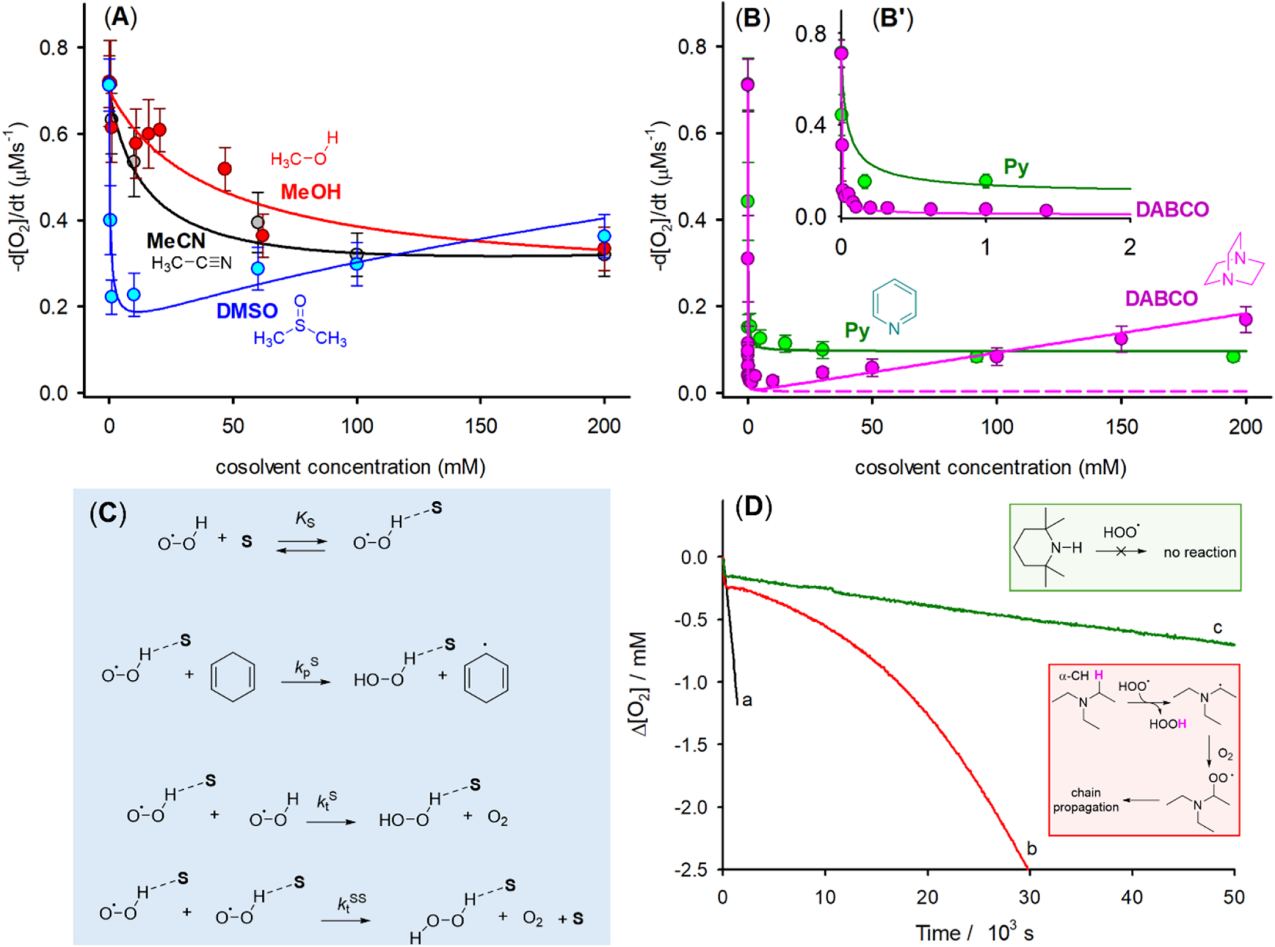
Rate of O_2_ consumption during the autoxidation of CHD 0.26 M in PhCl initiated by AIBN (25 mM) at 30 °C in the presence of increasing amounts of cosolvents (see Supporting Information for details); solid lines represent the result of the fitting by eqs 2 and 3 (see Figures S16–S20) (A, B); proposed mechanism for the kinetic solvent effect (KSE) due to cosolvents S (C); oxygen consumption during the autoxidation of CHD in PhCl in the absence of cosolvents (black line) or in the presence of triethylamine (red line) or TMP (green line), both 10 μM (D).

Interestingly, Figure 2A reveals that the concentration of cosolvent required to alter the rate of autoxidation depends on the hydrogen-bond-accepting (HBA) ability of S. This ability increases from MeCN (β_2_^H^ = 0.44) to DMSO (β_2_^H^ = 0.78),^39^ suggesting that the observed effects derive from one-to-one H-bond interactions and depend on the magnitude of the equilibrium constant *K*_S_. Methanol (β_2_^H^ = 0.41)^39^ is a solvent able to both accept and donate H-bonds. However, its effect on the oxidation rate of CHD is similar to that of MeCN (compare the red and black curves in Figure 2A). As MeOH and MeCN have very similar β_2_^H^ values, it can be concluded that at low concentrations MeOH behaves mainly as H-bond acceptor. The similarity in the MeOH and MeCN kinetics is also indirect proof that MeOH oxidation does not contribute to O_2_ consumption.

Regarding basic cosolvents, Py caused a dramatic reduction of CHD autoxidation rate (see green plot in Figure 2B) that remained unchanged with increasing concentration of the base. Similarly, DABCO displayed strong inhibitory activity, but differently from Py, when increasing DABCO concentrations beyond ca. 10 mM, an increase of −d­[O_2_]/d*t* was observed. We interpreted this result as effect of the participation of DABCO in the peroxidation chain, as it has six CH_2_ groups in α position to the N atoms that are susceptible to H atom abstraction from HOO^•^ radicals.^37^

To explore in a quantitative manner the effect of cosolvents on HOO^•^ reactivity, the data reported in Figure 2 were analyzed using eqs 1–3, which describe the rate of O_2_ consumption (−d­[O_2_]/d*t*) and KSE on the propagation and termination reaction, respectively (see Figure 2C).^40^

2 $$k_{p}=\frac{k_{p}^{0}+k_{p}^{S}K_{S}\left[S\right]}{1+K_{S}[S]}$$

3 $$k_{t}=\frac{k_{t}^{0}+k_{t}^{S}K_{S}\left[S\right]+k_{t}^{\mathrm{SS}}{\left(K_{S}\left[S\right]\right)}^{2}}{{\left(1+K_{S}\left[S\right]\right)}^{2}}$$

In eqs 2 and 3, *k*_p_ and *k*_t_ represent the apparent propagation and termination rate constants at a given cosolvent *S* concentration. *K*_S_ is the equilibrium constant for H-bond formation between HOO^•^ and S. The *k*_p_^0^ and *k*_t_^0^ constants are the propagation and termination reaction rate constants for “free” non-H-bonded HOO^•^, whose values in chlorobenzene are 1400 and 6.3 × 10^8^ M^–1^ s^–1^, respectively.^29^ *k*_p_^S^ is the propagation rate constant, and *k*_t_^S^ and *k*_t_^SS^ are the termination rate constants for the S···HOO^•^ H-bonded species (see Figure 2C).

The results of the fitting procedure are shown by the lines in Figure 2A,B and Table 1. We were gratified to find that eqs 1–3 can correctly reproduce KSE on the rate of oxidation, especially regarding the different effects exerted by DMSO at low and high concentration. The values of the equilibrium constant obtained from fitting (*K*_S_ (expt)) are in qualitative agreement with the β_2_^H^ values of the solvents, except for the relative order of the stronger acceptors DMSO, Py, and DABCO. In addition to showing large *K*_s_ values, DMSO, Py, and DABCO cause a dramatic decrease of *k*_p_^S^ values (see Table 1). Surprisingly, the effect of Py and DABCO on *k*_t_^SS^ was not as large as in case of DMSO and other nonbasic solvents, resulting in the overall inhibitory effect on CHD autoxidation.

**Table 1:** Experimental (Expt) Kinetic (M^–1^ s^–1^) and Equilibrium (M^–1^) Constants Obtained from the Fitting of Experimental Data to Equations 1–3.^a^ ^,^ ^b^

solvent^c^	β_2_^H^	*K*_S_ (expt)	*K*_S_ (calc)	*k*_p_^S^ (expt)	*k*_p_^S^ (calc)	*k*_t_^S^ (expt)	*k*_t_^S^ (calc)	*k*_t_^SS^ (expt)
PhCl/MeOH	0.41	50	43.3	90	67.9	7 × 10^8^	5.7 × 10^9^	0
PhCl/MeCN	0.44	70	34.1	130	55.4	9 × 10^8^	2.5 × 10^8^	0
PhCl/DMSO	0.78	2.0 × 10^3^	2 × 10^4^	20	9.1	4 × 10^8^	1.2 × 10^10^	0
PhCl/pyridine	0.62	3 × 10^5^	1.9 × 10^5^	5	1.5	1 × 10^8^	3.4 × 10^9^	3 × 10^5^
PhCl/DABCO	0.7^d^	3 × 10^6^	4.3 × 10^6^	0.1	0.75	6 × 10^8^	1.2 × 10^11^	2 × 10^5^

a Calculated (calc) values obtained from density functional theory modeling are also provided. β_2_^H^ values are from ref 39 and 45.

b Error of the fitting is estimated to be 30%.

c Kinetic constants calculated for the reaction: X + ^•^OOH···S → [X···^•^OOH···S]^‡^, where X = ^•^OOH or cyclohexadiene, using density functional theory with corrections for hydrogen atom tunneling (see text).

d Estimated value based on that of TEA.

We observed a steady increase of −d­[O_2_]/d*t* at high DABCO concentration (Figure 2B) and, as mentioned above, this observation is a consequence of the autoxidation of DABCO itself. In fact, the kinetic fitting without considering DABCO autoxidation was very poor, as reported in Figure 2B by the dashed line. Instead, we obtained a reasonable fitting of experimental data by assuming that the rate constant of the H atom transfer from DABCO to HOO^•^ is 10 M^–1^ s^–1^, a value that is similar to that of related amines.^38^ The effects of amine autoxidation were further investigated experimentally by measuring changes in [O_2_] over long reaction times (see Figure 2D). TEA, an amine that can be oxidized because it has α-CH_2_ groups, is seen to effectively inhibit CHD autoxidation for a time smaller than 3000 s, and this inhibition diminishes at longer reaction times. Radical oxidation of TEA is reported to give an imine^38^ that is far less basic than the parent amine.^41^ On the contrary, TMP showed a long lasting inhibition, which suggests that it is not consumed during the reaction. As it is known that the stable dialkylnitroxide TEMPO (2,2,6,6-tetramethyl-1-piperidinyloxyl) deriving from TMP is an excellent catalytic scavenger of HOO^•^,^42^ its formation was checked by EPR spectroscopy. However, analysis of the reaction mixture in the case of TMP did not show the formation of TEMPO (see Supporting Information, (Figure S21)). Based on this result and on the fact that we observe the inhibition of CHD autoxidation also in the presence of pyridine that does not form nitroxide radicals,^43^ the role of nitroxides could be excluded, in line with previous observations that conversion of TMP to TEMPO requires irradiation or high temperatures.^44^

From the *K*_S_(expt) reported in Table 1, the H-bond donating ability α_2_^H^ of HOO^•^ can be obtained from eq 4, and the known β_2_^H^ values of the cosolvents.^39,45^

4 $$\log(K_{\mathrm{HB}}M^{-1})=7.354\alpha_{2}^{H}\beta_{2}^{H}-1.094$$

The nonbasic cosolvents MeOH, MeCN and DMSO provide α_2_^H^ values for HOO^•^ in a close range, 0.87 ± 0.07, in agreement with previous estimates.^31^ Py and DABCO, the basic cosolvents, offer a substantially larger α_2_^H^ value (1.45 ± 0.02), which might be due to the proton transfer to the base to form a ionic couple: R_2_N···HOO^•^ → R_2_NH^+^···O_2_^•–^, a process that would shift toward the right of the H-bond equilibrium leading to an apparently higher *K*_S_.

To clarify the structure and the reactivity of the HOO^•^—solvent complexes, computational chemical modeling was performed using the CAM-B3LYP functional^46^ with aug-cc-pVTZ basis sets^47,48^ and the GD3­(BJ) empirical^49,50^ dispersion correction in continuum chlorobenzene solvent modeled via the SMD approach.^51^ The data obtained were used to determine rate constants via the Eyring equation. Tunneling corrections using an Eckart model were also included in the calculated *k*_p_^S^ and *k*_t_^S^ values. Preliminary calculations indicated that the ^•^OOHco-solvent complex exhibits a binary nature (see Figure S22). The results, which are reported in Table 1, are in good accord with the experimental values for all of the cosolvents. The equilibrium constants obtained for the S···HOO^•^ complex formation (*K*_s_) show excellent agreement with the values obtained from fitting the measured data. Likewise, the agreement between the fitted rate constants (*k*_p_ and *k*_t_^S^) and those obtained with our density functional theory (DFT) approach was excellent.

Further analysis of the computational data indicates that there are two interrelated factors associated with the increases in *k*_p_^S^ with increasing HBA strength, both of which are likely at play. Figure 3A plots the calculated free energy barriers associated with H-abstraction from CHD against the singly occupied molecular orbital energy level (ε­(SOMO)) in the S···HOO^•^ complexes (see Supporting Information, Table S2). As the strength of the hydrogen bond between HOO^•^ and the cosolvent increases, so too does the degree of charge transfer from S to the HOO^•^ moiety. The additional partial negative charge on HOO^•^ causes the SOMO, which is localized on HOO^•^, to be pushed to higher energies. The greater the energy separation between the SOMO and the relevant σ­(C–H) orbital in CHD, the weaker the interaction between them and the higher the free energy barrier for H-abstraction. As an aside, we note that all of the S···HOO^•^ complexes and HOO^•^ itself display SOMO-HOMO inversion. However, this phenomenon does not seem to play a critical role in the reaction kinetics for the S species investigated.

**Figure 3:**
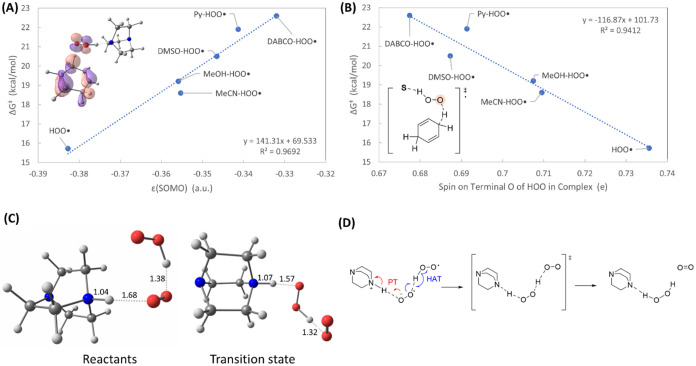
(A) Calculated free energy barriers for CHD abstraction by HOO^•^, with and without **S** complexation, versus the energy of the SOMO in **S**···HOO^•^. (B) Calculated free energy barriers for CHD abstraction by HOO^•^, with and without **S** complexation, versus the spin on the terminal oxygen atom of hydroperoxyl (highlighted in pink) in the transition state complex. (C) Calculated reactant complex and transition state structure for the termination reaction involving DABCO cosolvent. The bond lengths shown are in Å. Key: Gray = C, red = O, blue = N, white = H. (D) Proposed hydrogen atom and proton transfer (PT) mechanisms for the termination reaction with DABCO cosolvent.

Figure 3B is a plot of the calculated free energy barriers associated with H-abstraction from CHD versus the calculated excess spin density on the terminal oxygen in HOO^•^. The hydrogen bonding in S···HOO^•^ transfers β-spin charge into the σ*­(H–O) bond. The excess charge draws toward it α-spin from the terminal O atom, and this reduces the ability of that atom to abstract a hydrogen atom from CHD. Our DFT results did not reveal deprotonation of HOO^•^ in the H-bonded complexes formed with the bases in the solvent chlorobenzene.

The lengths of the **S---H**OO^•^ and **H–O**O^•^ bonds, shown in Table 2, are typical of noncovalent and covalent bonds, respectively, showing that no proton transfer occurs in chlorobenzene. A deeper look at the data in Table 2 revealed that the **S---H**OO^•^ distance is not always shorter for basic S (compare DMSO and Py in column 2), whereas the **H–O**O^•^ bond is longer for basic cosolvents, indicating an incipient proton transfer. The strong interaction between HOO^•^ and basic cosolvents causing the unusually high *K*_s_ can therefore be described in terms of charge separated resonance structures caused by the acidity of the HOO^•^ radical. Calculations also reveal that a 1:1 interaction between HOO^•^ and MeOH is adequate to explain its chemical behavior. In particular, the role of O atoms of HOO^•^ as H-bond acceptors is unimportant in the solvent under consideration, which is consistent with literature data on the low H-bond accepting capacity of ROO^•^.^15^

**Table 2:** Calculated Lengths (Å) of the Bonds Indicated in Bold; the Numbers in Parentheses Report the Difference between the TS and the Reactant

	S---HOO^•^		S---HOO^•^---CHD TS		S---HOO^•^--- HOO^•^ TS	
S	**S**---**H**OO^•^	S---**H–O**O^•^	**S**---**H**OO^•^	S---**H–O**O^•^	**S**---**H**OO^•^	S---**H–O**O^•^
-	-	0.98	-	0.97 (−0.01)	-	0.980 (+0.00)
MeCN	1.72	0.10	1.84 (+0.12)	0.98 (−0.02)	1.69 (−0.03)	1.002 (+0.00)
MeOH	1.61	1.01	1.72 (+0.11)	0.99 (−0.02)	1.58 (−0.04)	1.011 (+0.01)
DMSO	1.52	1.02	1.66 (+0.14)	0.99 (−0.03)	1.48 (−0.04)	1.033 (+0.01)
pyridine	1.58	1.04	1.72 (+0.14)	1.00 (−0.04)	1.52 (−0.06)	1.058 (+0.02)
DABCO	1.49	1.07	1.73 (+0.24)	0.98 (−0.10)	1.07 (−0.43)	1.565 (+0.49)^a^

a The large changes of bond lengths are due to proton transfer to DABCO.

Regarding the effect of H-bonds on HAT from CHD, Table 2 shows that the **S---H**OO^•^ distance becomes longer in going from the reactants to the TS, clearly indicating that the strength of the interaction decreases in the TS relative to that in the reactants. On the contrary, bond lengths remain almost unchanged in the TS of disproportionation, reasonably, because it is a strongly exergonic reaction with an “early” TS. The only exception is in the termination reaction involving DABCO, i.e., DABCO···HOO^•^ + HOO^•^. On the reactant side, our calculations indicate the formation of a charge transfer complex, wherein the HOO^•^ species H-bonded to DABCO transfers the proton to the R_3_N group (see Figure 3C). The O_2_^•–^ thus formed is stabilized by H-bond donation by the second HOO^•^ radical (Figure 3D). Interestingly, in the transition state (TS) in which the “outer” HOO^•^ transfers a H atom to the “inner” O^_2_•-^, protonated DABCO releases back the proton to the incipient H_2_O_2_ species, as can be inferred from the distances reported in Figure 3C. The character of this TS structure suggests that the termination mechanism with this cosolvent base is a proton-coupled hydrogen transfer, as schematized in Figure 3D.

Despite numerous attempts, we could not find any TS for the disproportionation of two S···HOO^•^ species (i.e., the reaction relative to the rate constant *k*_t_^SS^) when S is a basic cosolvent. It may be suggested that this reaction proceeds through proton-coupled electron transfer (PCET), greatly facilitated by the H-bond with the base,^22^ followed by a fast proton transfer (eq 5).

$$2\,\;S\cdot \cdot \cdot {\mathrm{HOO}}^{•}\overset{\mathrm{PCET}}{\to }{\mathrm{SH}}^{+}+O_{2}+S\cdot \cdot \cdot {\mathrm{HOO}}^{-}\overset{\mathrm{PT}}{\to }S+O_{2}+H_{2}O_{2}$$ 5

### High-Polarity Medium

The effect of bases on CHD autoxidation was then investigated experimentally by using acetonitrile (MeCN) as the main solvent. Being more polar than chlorobenzene, MeCN can be expected to support the complete deprotonation of HOO^•^ with a cosolvent base of sufficient strength. The results reported in Figure 4A confirmed our preliminary observations^52^ and showed that the O_2_ consumption rate is reduced in proportion to the concentration and strength of the base added, as measured by the p*K*_a_ of the conjugate acid in MeCN (Table S3 and Figures S23–S27). Pyridine, the weakest base with p*K*_a_^MeCN^ = 12.5,^53^ caused only a relatively small reduction of O_2_ consumption. Tert-octylamine (1,1,3,3-tetramethylbutylamine, p*K*_a_^MeCN^ = 18),^54^ piperidine (p*K*_a_^MeCN^ = 19.4),^54^ and tetramethylpiperidine (p*K*_a_^MeCN^ = 19.9)^55^ showed large reductions in O_2_ consumption, reaching a plateau of virtually no O_2_ consumption at base concentrations of ca. 1 mM. The very strong base Bu_4_NOH (p*K*_a_^MeCN^ ≈ 21.6)^55^ completely arrested CHD autoxidation at the smallest concentration used (0.1 mM). These results also allow us to place the p*K*_a_^MeCN^ of HOO^•^ in MeCN between 18 and 19. These results strongly indicate that piperidine, tetramethylpiperidine, and Bu_4_NOH completely deprotonate HOO^•^. DFT optimizations of the piperidine-HOO^•^ complex in an acetonitrile implicit solvent also demonstrate that proton transfer occurs (Figure S28 and Table S4).

**Figure 4:**
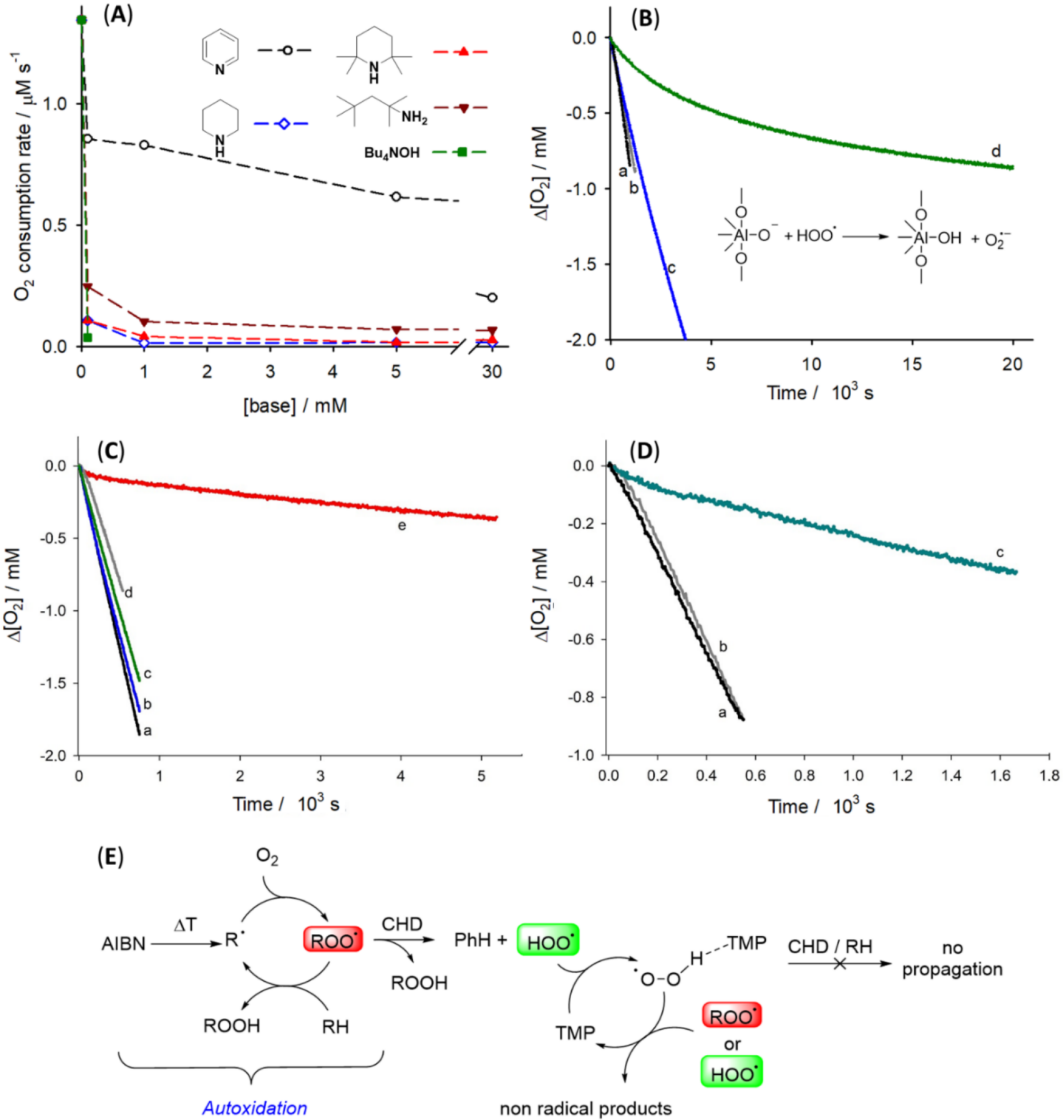
O_2_ consumption rate during the autoxidation of CHD 0.26 M initiated by AIBN (25 mM) in MeCN at 30 °C, in the presence of increasing amounts of bases, dashed lines are guides for the eyes (A); O_2_ consumption during the autoxidation of CHD 0.26 M in MeCN (line a) or PhCl (line b) without antioxidants or in the presence of activated alumina (12.5 mg/mL) (line c in PhCl and line d in MeCN) (B); Oxygen consumption during the autoxidation of styrene (4.3 M) initiated by AIBN (50 mM) at 30 °C in PhCl (a) or in the presence of TMP 9 μM (b) or 27 μM (c), CHD 0.26 M (d), TMP 9 μM, and CHD 0.26 M (e) (C); oxygen consumption during the autoxidation of styrene (4.3 M) initiated by AIBN (50 mM) at 30 °C in PhCl (a) or in the presence of pyridine 60 mM (b), pyridine 60 mM, and CHD (0.26 M) (c) (D); mechanism involved in the catalytic antioxidant effect (E).

### Heterogeneous Bases

Intrigued by the dramatic effects of bases on CHD autoxidation, we decided to explore the possibility that similar effects would be observed in a heterogeneous system consisting of basic materials finely dispersed in the reaction medium. For this purpose, we employed activated basic alumina (Brockmann I), a powder with particles having a diameter ≈ 100 μm and 58 Å pores commonly used as stationary phase for column purification.^56^ The powder was well dispersed during the reaction by vigorous stirring with a magnetic stir bar. The results, reported in Figure 4B, showed that alumina retards CHD autoxidation in acetonitrile, whereas no effect was seen in chlorobenzene, presumably because the surface of the material is not “wetted” by the solvent. This result demonstrates that heterogeneous bases in appropriate solvents can reduce the HOO^•^ reactivity in a manner similar to that observed with basic solvents.

### Implication in Antioxidant Defense

Keeping organic materials under O_2_ inevitably causes their degradation through a radical-chain mechanism named autoxidation, or peroxidation, with the formation of a series of toxic and/or bad-smelling oxygenated derivatives.^57^ Developing strategies for retarding autoxidation is of fundamental importance, for example, to prevent rancidity of fat rich foods (like edible oils), or to preserve lubricating oil or plastic.^58^ While the radicals that sustain the autoxidation radical chain are often represented by alkylperoxyl radicals (ROO^•^), in some notable cases like primary and secondary alcohols, amines, and others of potential biological and technological relevance, the HOO^•^ radical is also formed as a result of ROO^•^ fragmentation.^59^ As a consequence, the contemporary presence of ROO^•^ and HOO^•^ in autoxidation and in other radical chains of practical relevance is of great interest, although often undervalued. We have previously shown^33^ that the presence of mixed ROO^•^/HOO^•^ radicals can be easily achieved by mixing CHD with an oxidizable substrate which would autoxidise only through ROO^•^. In this way, ROO^•^ radicals will react with CHD to form HOO^•^, which, in turn, can either propagate the chain by reacting with the substrate (RH) or be trapped by antioxidants.

As a proof-of-concept, a nonoxidizable basic cosolvent was added to an autoxidizing system where ROO^•^ and HOO^•^ radicals were contemporarily present. Figure 4C,D shows O_2_ consumption measured during the autoxidation of a model substrate (styrene)^33^ initiated by AIBN. The addition of CHD did not modify the oxidation rate of styrene, nor did the addition of TMP at two different concentrations (see Figure 4C traces a–d). However, the simultaneous presence of CHD and TMP caused a marked inhibition of the oxidation of styrene (Figure 4C trace e) over a long time period, in line with the results presented about showing that TMP is not consumed during the reaction. The same effect is present by adding pyridine as a base into the reaction environment, but in this case, inhibition is achieved at a much higher concentration (60 mM vs 9 μM for Py and TMP, respectively). We can conclude that TMP and pyridine behave as “catalytic” antioxidants, by engaging in strong hydrogen bonding with HOO^•^ and thereby increasing the barrier associated with propagation. Under these conditions, these bases do not inhibit the ability of HOO^•^ to react with itself to terminate the chain. The proposed mechanism is reported in Figure 4E.

## Conclusions

The study of the radical-chain autoxidation kinetics of 1,4-cyclohexadiene provided valuable insights into the behavior of the HOO^•^ radical. In the moderately apolar solvent chlorobenzene, the reactivity of HOO^•^ was finely tuned by a series of cosolvents with different hydrogen-bond accepting and basic properties. This allowed for a predictive understanding of the radical’s hydrogen-bonding behavior across various conditions. For the first time, we demonstrated that basic cosolvents strongly bind to the HOO^•^ radical, significantly diminishing its ability to abstract hydrogen atoms, while simultaneously facilitating its disproportionation. The antioxidant activity of basic molecules or solids in the presence of HOO^•^, when the key chain-carrying radical species are in autoxidation, offers new strategies for mitigating the autoxidation of organic compounds. Previously, it was believed that trapping HOO^•^ radicals required a redox-active agent capable of cycling between reduced and oxidized states, thereby promoting HOO^•^ disproportionation (e.g., TEMPO).^33^ However, our findings suggest that basic substances can also effectively modulate the HOO^•^ reactivity, opening up alternative approaches for controlling autoxidation without relying solely on traditional redox-active species. The success of the base-catalyzed antioxidant effect is determined by the oxidizable substrate’s (i.e., the molecules to be protected) ability to produce HOO^•^ radicals during autoxidation. Adding chain-transfer agents, such as CHD derivatives like γ-terpinene, offers a promising way to utilize this chemistry. The nonoxidizable amine base TMP demonstrated exceptionally extended antioxidant protection, which was triggered by an unusual catalytic cycle based on the TMP---HOO^•^ complex. Furthermore, we showed that finely dispersed basic materials share this property, paving the way for the rational design of solid oxidation inhibitors and the use of nanomaterials with antioxidant properties. We believe that these findings will be extremely useful in the rational design of diverse, highly effective antioxidant systems for stabilizing easily oxidizable components in food, plastics, and lubricants.

## Experimental Section

Experimental procedures and data are given in the Supporting Information.

## Supplementary Material





## Data Availability

The data underlying this study are available in the published article and its Supporting Information.

## Notes

Experimental procedures, calculation details (PDF)
Optimized geometries (ZIP)
