# Supplementary material for: Solvent Effects on C–H Abstraction by Hydroperoxyl Radicals: Implication for Antioxidant Strategies
Source: J Org Chem. 2025 Sep 15;90(38):13467–76. doi: 10.1021/acs.joc.5c01140 (PMC12481551; doi:10.1021/acs.joc.5c01140)
Supplement: Supplementary file 1 [file jo5c01140_si_001.pdf]

Supplementary Information

Solvent Effects on C-H Abstraction by Hydroperoxyl Radicals: Implication for Antioxidant Strategies

Andrea Baschieri,*[a] Zongxin Jin,[b] Greta Tödtmann,[c] Gino A. DiLabio,*[c] Riccardo Amorati*[b]

[a] *Institute for Organic Synthesis and Photoreactivity (ISOF), National Research Council of Italy (CNR), Via Piero Gobetti 101, 40129 Bologna, Italy.*

[b] *Department of Chemistry "G. Ciamician", University of Bologna, Via Gobetti 83, 40129 Bologna, Italy*

[c] *Department of Chemistry, The University of British Columbia, 3247 University Way, Kelowna, British Columbia, Canada V1V 1V7.*

Table of contents

Content	Page
Experimental procedures	S2
Slope of rate of O_2 consumption during the autoxidation of CHD in the presence of increasing amounts of co-solvents (PhCl).	S3 - S10
R_i variation after MeCN additions	S11
Fitting of experimental data reported in Figure 1	S11 – S13
EPR spectra of TMP	S14
Optimization of the $HOO^{\bullet}$ radical with five acetonitrile molecules	S14
Free energy data of the investigated species	S15
Slope of rate of O_2 consumption during the autoxidation of CHD in the presence of increasing amounts of bases (ACN).	S15 – S18
DFT optimizations of the piperidine-$HOO^{\bullet}$ complex	S18
Optimized coordinates for the piperidine-HOO complex	S19
References	S20

Experimental procedures

Materials. All solvents were of the highest grade commercially available (≥99.9% HPLC grade) and used as received. All chemicals were of reagent grade and were used without further purification. 1,4 cyclohexadiene and styrene were purified by double percolation through silica and activated alumina columns in order to remove traces of stabilizer. 2,2-azobis(2-methylpropionitrile) (AIBN) was recrystallized from methanol and stored at -18 °C.

Inhibited Autoxidation Studies. Autoxidation experiments were performed by measuring the oxygen consumption in a two-channel gas uptake apparatus, immersed in a thermostated bath, based on Validyne DP15 pressure transducer. The rate of initiation (R_i) was calculated from preliminary set of experiments from the length of the inhibition period, τ, using 2,2,5,7,8-pentamethyl-6-chromanol (PMHC) as a reference antioxidant during autoxidation of styrene, $R_i = 2[\text{PMHC}]/\tau$. PhCl /Styrene R_i = 6.2×10^{-9} Ms^{-1}. The $2k_t$ value of styrene at 303 K is 4.2×10^{7} $M^{-1}s^{-1}$.[1] In a typical experiment, an air-saturated solution of CHD 0.26 M containing AIBN at 30 °C was equilibrated with an identical reference solution containing excess 2,2,5,7,8-pentamethyl-6-hydroxychromane (PMHC) (25 mM). After equilibration, and when a constant O_2 consumption was reached, co-solvent or the base was injected in the sample flask. After approximately 1 hour the experiment is stopped and the slope relating to oxygen consumption in those specific conditions is determined. This operation is carried out initially without adding bases or co-solvents and after their addition at different concentrations.

Fittings reported in Figure 2 were performed by using the software LabPlot Version 2.11.1, freely available at the webpage: https://labplot.kde.org.

The error of oxygen consumption ($-d[O_2]/dt$) is within 20%.

Computational details. All quantum chemical calculations employed the CAM-B3LYP[2] density functional with aug-cc-pVTZ basis sets.[3,4] The D3 dispersion correction[5] with the Becke-Johnson damping function[6] was applied throughout. The effects of chlorobenzene solvent were approximated using the SMD implicit solvent model.[7] Optimized ground state structures were verified to have all positive vibration frequencies. All transition state structures were verified to possess one imaginary vibration mode that connects reactants to products. All calculations were performed using the Gaussian 16 program.[8] The data used to calculate the energies, equilibrium and rate constants are reported in a separate .xlsx file. Cartesian coordinates (.xyz files) are reported in a separate compressed folder (optimized structures.zip).

EPR experiments. The EPR spectra were collected at 25 °C with a MiniScope MS 5000 (Magnettech).

Table S1. Slope of rate of O_2 consumption during the autoxidation of CHD 0.26 M in PhCl initiated by AIBN (25 mM) at 30 °C in the presence of increasing amounts of co-solvents.

Conc (M) **ACN**	slope $-d[O_2]/dt$ (M s^{-1}) **ACN**	Conc (M) **MeOH**	slope $-d[O_2]/dt$ (M s^{-1}) **MeOH**	Con (M) **DMSO**	slope $-d[O_2]/dt$ (M s^{-1}) **DMSO**
0	7.13E-07 ± 6.0E-8	0	7.21E-07 ± 6.0 E -8	0	7.13E-07 ± 6.0E-8
1.00E-03	6.33E-07 ± 8.0E-8	1.00E-04	7.17E-07 ± 1.0 E -7	1.00E-03	2.22E-07 ± 8.0E-8
0.01	5.35E-07 ± 8.0E-8	6.40E-04	7.17E-07 ± 1.0 E -7	0.01	2.27E-07 ± 4.0E-8
0.06	3.95E-07 ± 7.0E-8	1.00E-03	6.14E-07 ± 8.0 E -8	0.06	2.88E-07 ± 5.0E-8
0.1	3.20E-07 ± 5.0E-8	0.0108	5.78E-07 ± 8.0 E -8	0.1	2.98E-07 ± 5.0E-8
0.2	3.20E-07 ± 5.0E-8	0.0159	6.00E-07 ± 8.0 E -8	0.2	3.63E-07 ± 5.0E-8
		0.0204	6.09E-07 ± 5.0 E -8		
		0.0468	5.18E-07 ± 5.0 E -8		
		0.062	3.64E-07 ± 5.0 E -8		
		0.2	3.34E-07 ± 5.0 E -8		

Conc (M) **Py**	slope $-d[O_2]/dt$ (M s^{-1}) **Py**	Conc (M) **DABCO**	slope $-d[O_2]/dt$ (M s^{-1}) **DABCO**
0	7.13e-7 ± 6.0e-8	0	7.10E-07 ± 6.0e-8
1.5000e-6	4.43e-7 ± 9.0e-8	5.00E-06	3.10E-07 ± 1.0e-7
1.6250e-4	1.51e-7 ± 3.0e-8	1.00E-05	1.14E-07 ± 4.0e-8
1.0000e-3	1.54e-7 ± 3.0e-8	2.50E-05	8.80E-08 ± 4.0e-9
5.0015e-3	1.26e-7 ± 2.0e-8	5.00E-05	9.69E-08 ± 2.0e-8
0.0150	1.14e-7 ± 2.0e-8	8.00E-05	6.19E-08 ± 6.0e-9
0.0300	9.92e-8± 2.0e-8	1.00E-04	4.10E-08 ± 1.0e-8
0.0919	8.27e-8± 1.0e-8	2.00E-04	3.73E-08 ± 6.0e-9
0.1950	8.32e-8 ± 1.0e-8	3.20E-04	3.65E-08 ± 6.0e-9
		6.20E-04	2.98E-08 ± 1.0e-8
		1.00E-03	2.99E-08 ± 8.0e-9
		1.42E-03	2.43E-08 ± 8.0e-9
		3.00E-03	3.88E-08 ± 8.0e-9
		0.01	2.73E-08 ± 1.0e-8
		0.03	4.70E-08 ± 1.0e-8
		0.05	5.79E-08 ± 2.0e-8
		0.1	8.36E-08 ± 2.0e-8
		0.15	1.25E-07 ± 3.0e-8
		0.2	1.69E-07 ± 3.0e-8

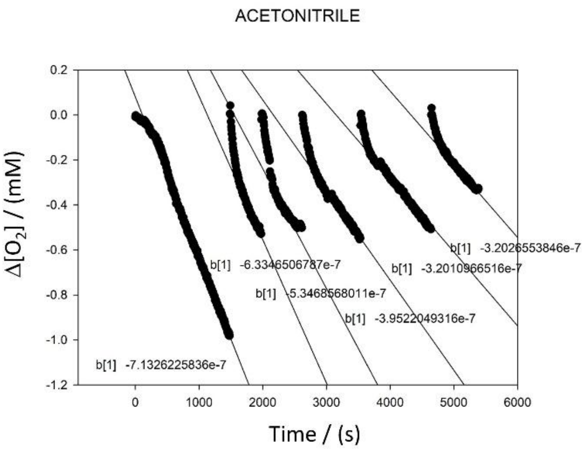

Figure S1. O_2 consumption during the autoxidation of CHD 0.26 M in PhCl initiated by AIBN (25 mM) at 30 °C in the presence of increasing amounts of co-solvents.

Sample: 2 mL AIBN in PhCl 0,05 M + 1,9 mL PhCl + 100 μL CHD

+ 5 μL ACN 1,28 M in PhCl (final concentration = 1,6 mM)

+ 30 μL ACN 1,28 M (final concentration = 11,6 mM)

+ 10 μL ACN pure (final concentration = 59,5 mM)

+ 10 μL ACN pure (final concentration = 107,4 mM)

+ 20 μL ACN pure (final concentration = 203,4 mM)

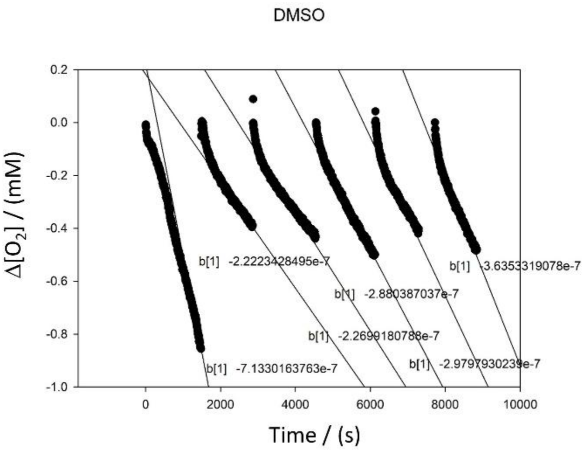

Figure S2. O_2 consumption during the autoxidation of CHD 0.26 M in PhCl initiated by AIBN (25 mM) at 30 °C in the presence of increasing amounts of co-solvents.

Sample: 2 mL AIBN in PhCl 0,05 M + 1,9 mL PhCl + 100 μL CHD

+ 5 μL DMSO 1,13 M in PhCl (final concentration = 1,4 mM)

+ 35 μL DMSO 1,13 M (final concentration = 11,3 mM)

+ 15 μL DMSO pure (final concentration = 64,3 mM)

+ 15 μL DMSO pure (final concentration = 117,3 mM)

+ 30 μL DMSO pure (final concentration = 223,3 mM)

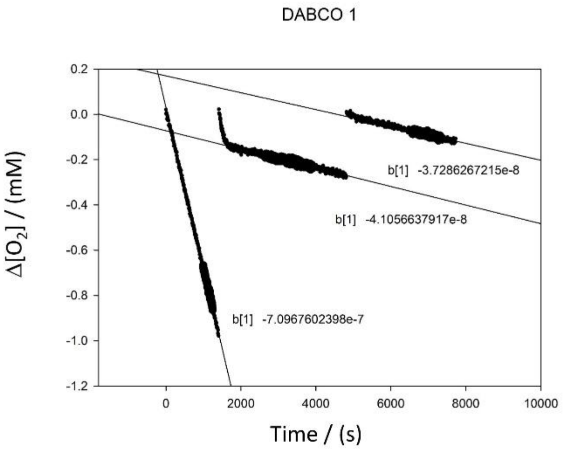

Figure S3. O_2 consumption during the autoxidation of CHD 0.26 M in PhCl initiated by AIBN (25 mM) at 30 °C in the presence of increasing amounts of co-solvents.

Sample: 2 mL AIBN in PhCl 0,05 M + 1,9 mL PhCl + 100 μL CHD

+ 10 μL DABCO 40 mM in PhCl (final concentration = 0.1 mM)

+ 10 μL DABCO 40 mM in PhCl (final concentration = 0.2 mM)

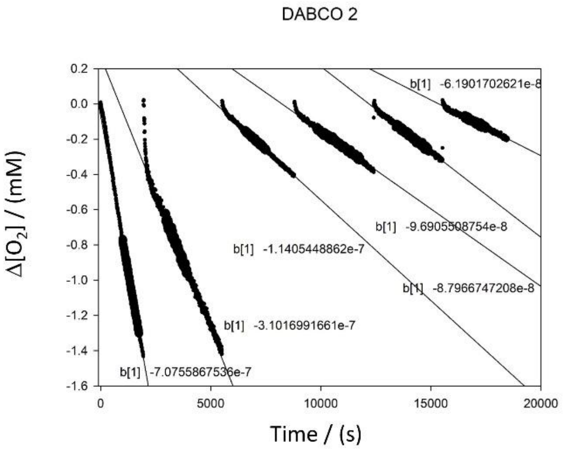

Figure S4. O_2 consumption during the autoxidation of CHD 0.26 M in PhCl initiated by AIBN (25 mM) at 30 °C in the presence of increasing amounts of co-solvents.

Sample: 2 mL AIBN in PhCl 0,05 M + 1,9 mL PhCl + 100 μL CHD

+ 5 μL DABCO 4 mM in PhCl (final concentration = 0.005 mM)

+ 5 μL DABCO 4 mM in PhCl (final concentration = 0.01 mM)

+ 15 μL DABCO 4 mM in PhCl (final concentration = 0.025 mM)

+ 25 μL DABCO 4 mM in PhCl (final concentration = 0.05 mM)

+ 30 μL DABCO 4 mM in PhCl (final concentration = 0.08 mM)

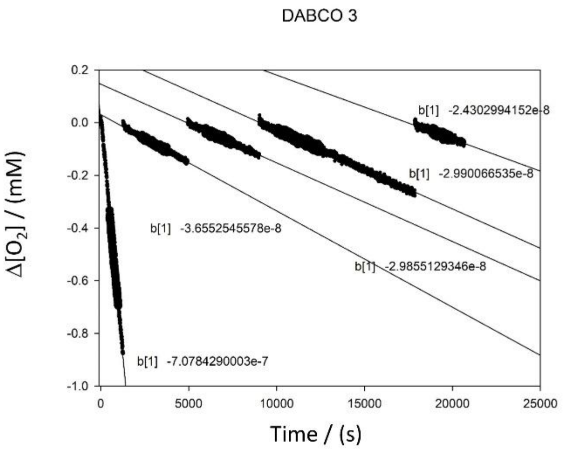

Figure S5. O_2 consumption during the autoxidation of CHD 0.26 M in PhCl initiated by AIBN (25 mM) at 30 °C in the presence of increasing amounts of co-solvents.

Sample: 2 mL AIBN in PhCl 0,05 M + 1,9 mL PhCl + 100 μL CHD

+ 32 μL DABCO 40 mM in PhCl (final concentration = 0.32 mM)

+ 30 μL DABCO 40 mM in PhCl (final concentration = 0.62 mM)

+ 10 μL DABCO 160 mM in PhCl (final concentration = 1.02 mM)

+ 10 μL DABCO 160 mM in PhCl (final concentration = 1.42 mM)

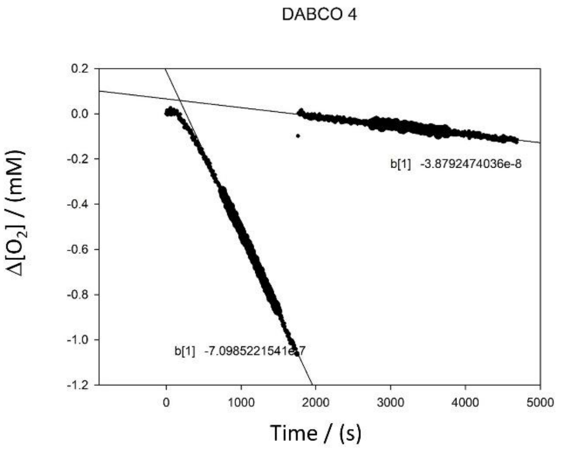

Figure S6. O_2 consumption during the autoxidation of CHD 0.26 M in PhCl initiated by AIBN (25 mM) at 30 °C in the presence of increasing amounts of co-solvents.

Sample: 2 mL AIBN in PhCl 0,05 M + 1,9 mL PhCl + 100 μL CHD

+ 30 μL DABCO 400 mM in PhCl (final concentration = 3 mM)

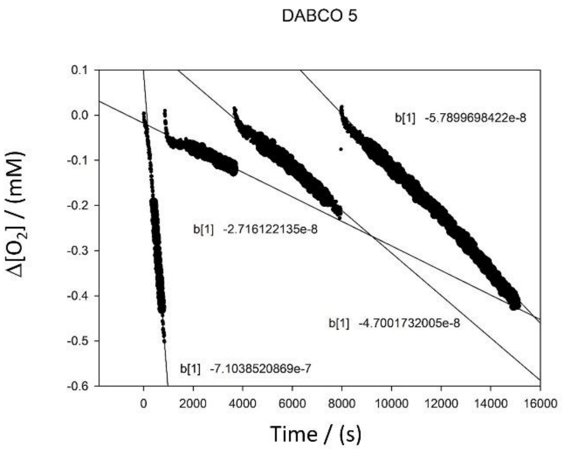

Figure S7. O_2 consumption during the autoxidation of CHD 0.26 M in PhCl initiated by AIBN (25 mM) at 30 °C in the presence of increasing amounts of co-solvents.

Sample: 2 mL AIBN in PhCl 0,05 M + 1,9 mL PhCl + 100 μL CHD

+ 50 μL DABCO 800 mM in PhCl (final concentration = 10 mM)

+ 100 μL DABCO 800 mM in PhCl (final concentration = 30 mM)

+ 100 μL DABCO 800 mM in PhCl (final concentration = 50 mM)

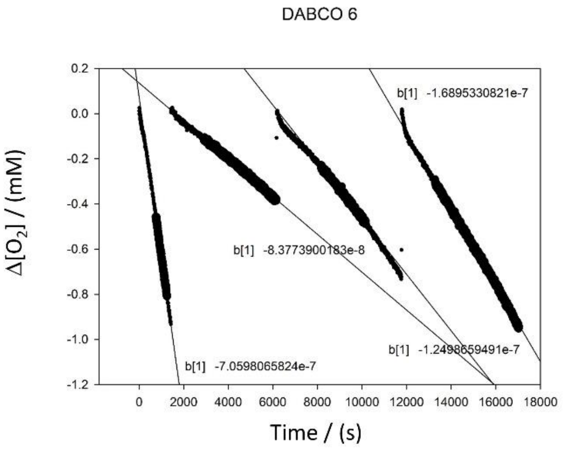

Figure S8. O_2 consumption during the autoxidation of CHD 0.26 M in PhCl initiated by AIBN (25 mM) at 30 °C in the presence of increasing amounts of co-solvents.

Sample: 2 mL AIBN in PhCl 0,05 M + 1,9 mL PhCl + 100 μL CHD

+ 160 μL DABCO 2.5 M in PhCl (final concentration = 100 mM)

+ 80 μL DABCO 2.5 M in PhCl (final concentration = 150 mM)

+ 80 μL DABCO 2.5 M in PhCl (final concentration = 200 mM)

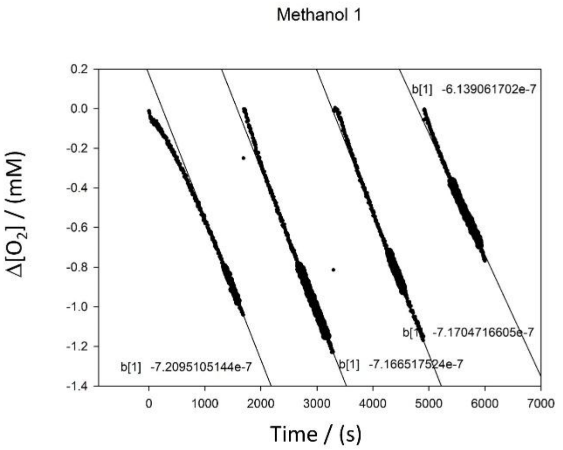

Figure S9. O_2 consumption during the autoxidation of CHD 0.26 M in PhCl initiated by AIBN (25 mM) at 30 °C in the presence of increasing amounts of co-solvents.

Sample: 2 mL AIBN in PhCl 0,05 M + 1,9 mL PhCl + 100 μL CHD

+ 20 μL MeOH 0.021 M in PhCl (final concentration = 0.1 mM)

+ 100 μL MeOH 0.021 M in PhCl (final concentration = 0.63 mM)

+ 80 μL MeOH 0.021 M in PhCl (final concentration = 1 mM)

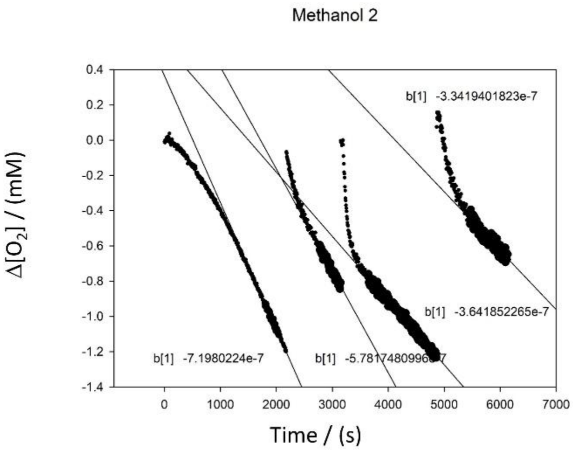

Figure S10. O_2 consumption during the autoxidation of CHD 0.26 M in PhCl initiated by AIBN (25 mM) at 30 °C in the presence of increasing amounts of co-solvents.

Sample: 2 mL AIBN in PhCl 0,05 M + 1,9 mL PhCl + 100 μL CHD

+ 40 μL MeOH 1.03 M in PhCl (final concentration = 10.3 mM)

+ 8 μL MeOH pue in PhCl (final concentration = 60.3 mM)

+ 24 μL MeOH pue in PhCl (final concentration = 210 mM)

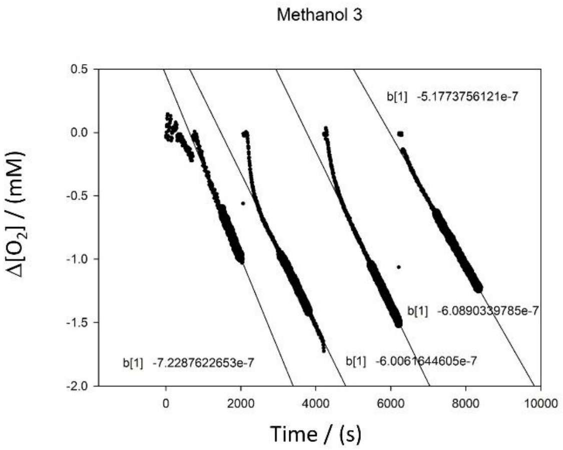

Figure S11. O_2 consumption during the autoxidation of CHD 0.26 M in PhCl initiated by AIBN (25 mM) at 30 °C in the presence of increasing amounts of co-solvents.

Sample: 2 mL AIBN in PhCl 0,05 M + 1,9 mL PhCl + 100 μL CHD

+ 30 μL MeOH 2.06 M in PhCl (final concentration = 15 mM)

+ 10 μL MeOH 2.06 M in PhCl (final concentration = 20 mM)

+ 10 μL MeOH 2.06 M in PhCl (final concentration = 20 mM)

+ 25 μL MeOH 4.12 M in PhCl (final concentration = 46 mM)

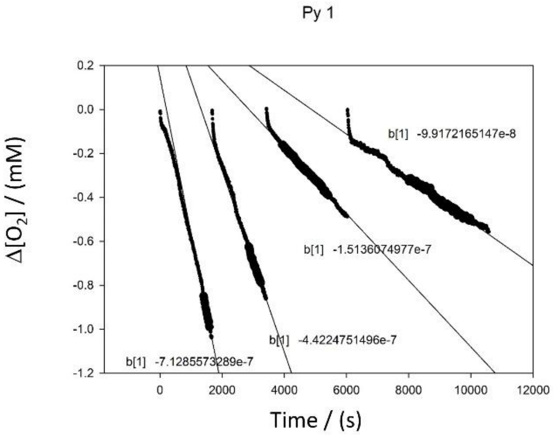

Figure S12. O_2 consumption during the autoxidation of CHD 0.26 M in PhCl initiated by AIBN (25 mM) at 30 °C in the presence of increasing amounts of co-solvents.

Sample: 2 mL AIBN in PhCl 0,05 M + 1,9 mL PhCl + 100 μL CHD

+ 6 μL Py 1 mM in PhCl (final concentration = 0.0015 mM)

+ 22 μL Py 30 mM in PhCl (final concentration = 0.16 mM)

+ 10 μL Py pure in PhCl (final concentration = 30 mM)

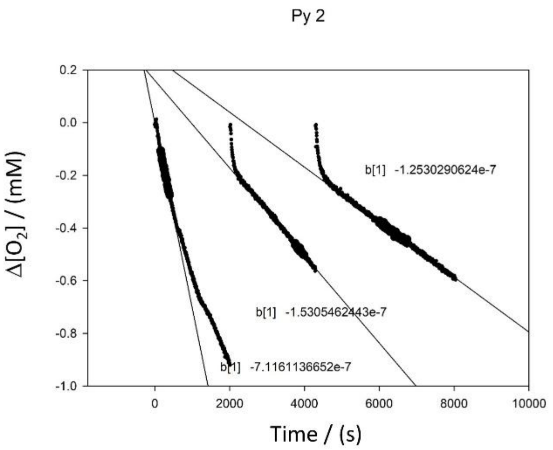

Figure S13. O_2 consumption during the autoxidation of CHD 0.26 M in PhCl initiated by AIBN (25 mM) at 30 °C in the presence of increasing amounts of co-solvents.

Sample: 2 mL AIBN in PhCl 0,05 M + 1,9 mL PhCl + 100 μL CHD

+ 20 μL Py 0.25 M in PhCl (final concentration = 1 mM)

+ 16 μL Py 1 M in PhCl (final concentration = 5 mM)

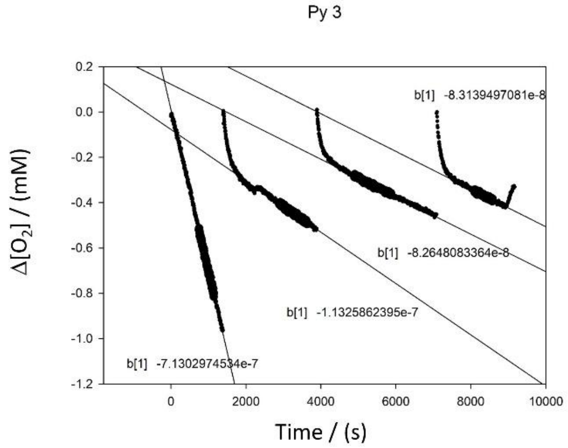

Figure S14. O_2 consumption during the autoxidation of CHD 0.26 M in PhCl initiated by AIBN (25 mM) at 30 °C in the presence of increasing amounts of co-solvents.

Sample: 2 mL AIBN in PhCl 0,05 M + 1,9 mL PhCl + 100 μL CHD

+ 30 μL Py 2 M in PhCl (final concentration = 15 mM)

+ 25 μL Py pure in PhCl (final concentration = 90 mM)

+ 35 μL Py pure in PhCl (final concentration = 195 mM)

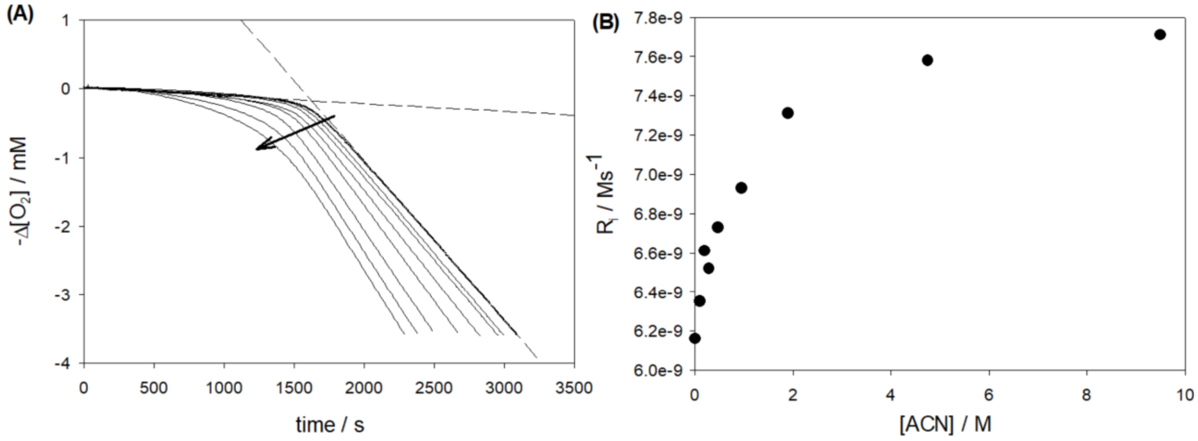

Figure S15. A) O_2 consumption traces recorded during the autoxidation of styrene (4.3 M) in PhCl initiated by AIBN (0.05 M) at 30 °C in the presence of α-tocopherol (α-TOH, 5.0 μM) at increasing MeCN concentrations (M): 0, 0.19, 0.29, 0.47, 0.95, 1.9, 4.8, 9.5. The crossing point of the regression lines at the inhibited and not inhibited period is used to measure τ. B) R_i values calculated from the length of the inhibited period (t) and the equation: R_i=2[α-TOH]/τ.

Figure S16. Fitting of experimental data reported in Figure 1 for DABCO. Coefficient of determination (R^2) = 0.931208.

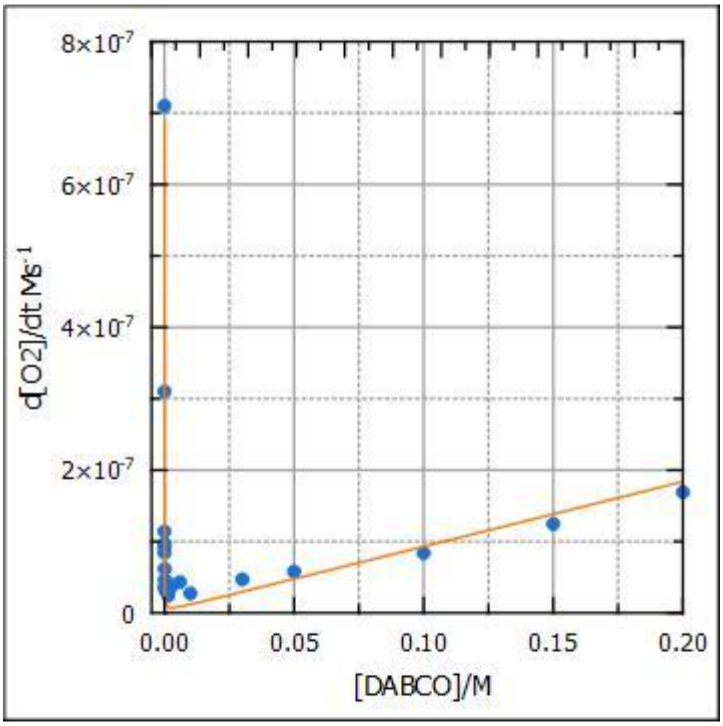

Figure S17. Fitting of experimental data reported in Figure 1 for DMSO. Coefficient of determination (R^2) = 0.910378.

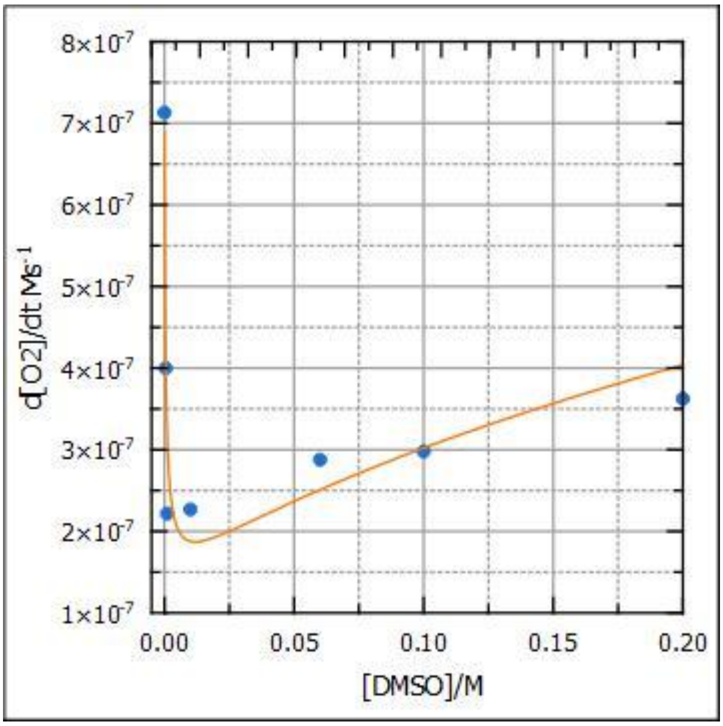

Figure S18. Fitting of experimental data reported in Figure 1 for acetonitrile. Coefficient of determination (R^2) = 0.962969.

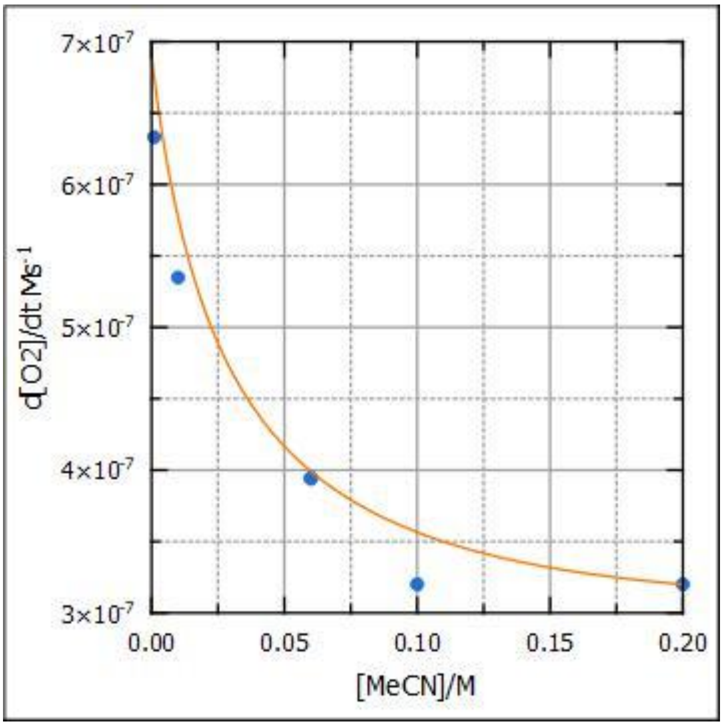

Figure S19. Fitting of experimental data reported in Figure 1 for methanol. Coefficient of determination (R^2) = 0.75955.

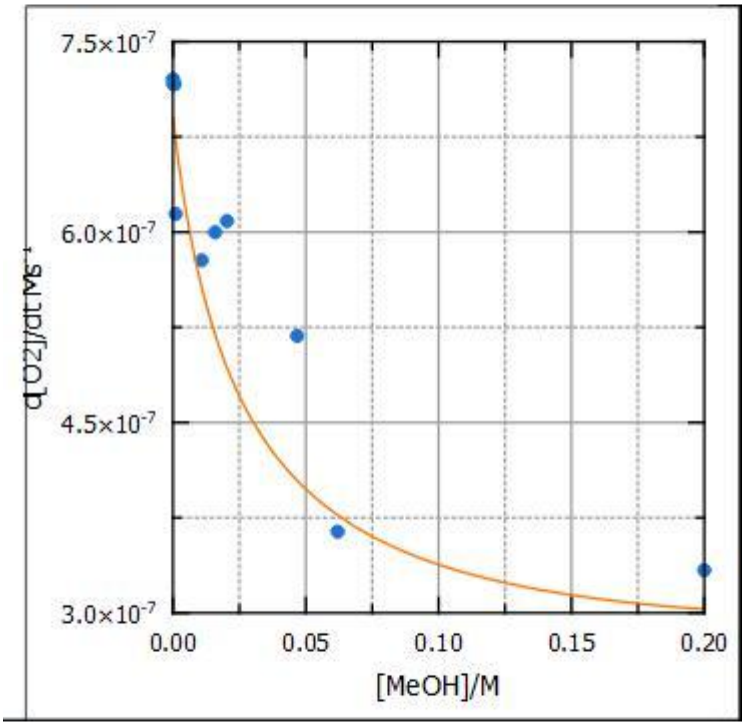

Figure S20. Fitting of experimental data reported in Figure 1 for pyridine. Coefficient of determination (R^2) = 0.836194.

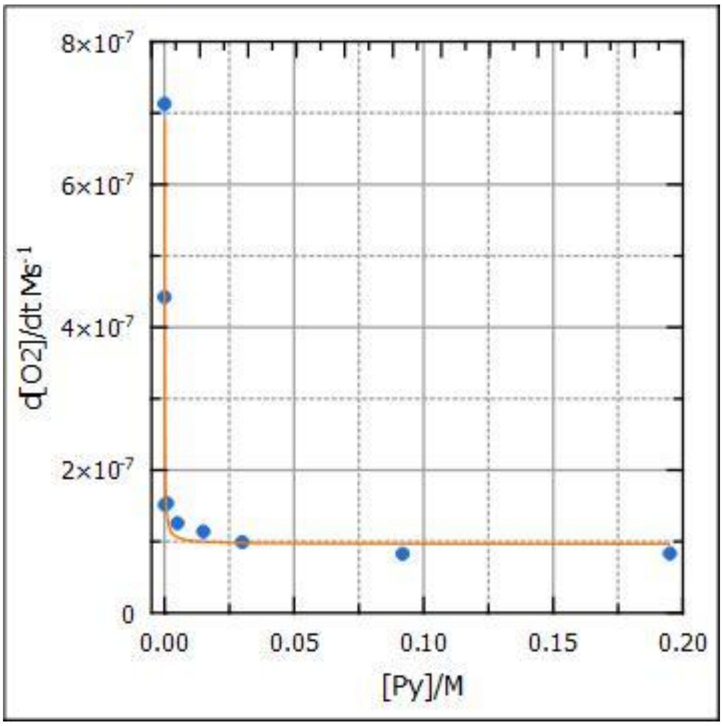

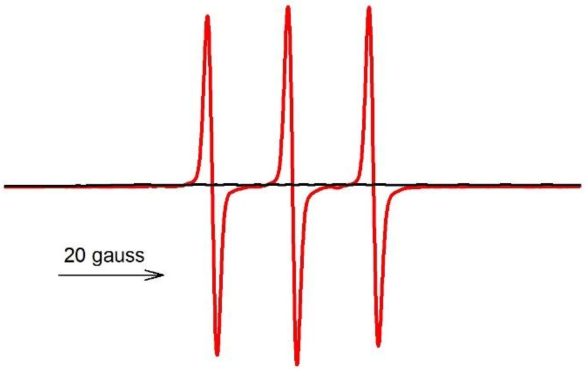

Figure S21. Black line: EPR spectra of the reaction mixture containing CHD (0.26 M), AIBN (25 mM) and TMP 10 uM in PhCl after 2 h of reaction at 30 °C. **Red line**: reference spectrum of TEMPO (10 uM) in PhCl.

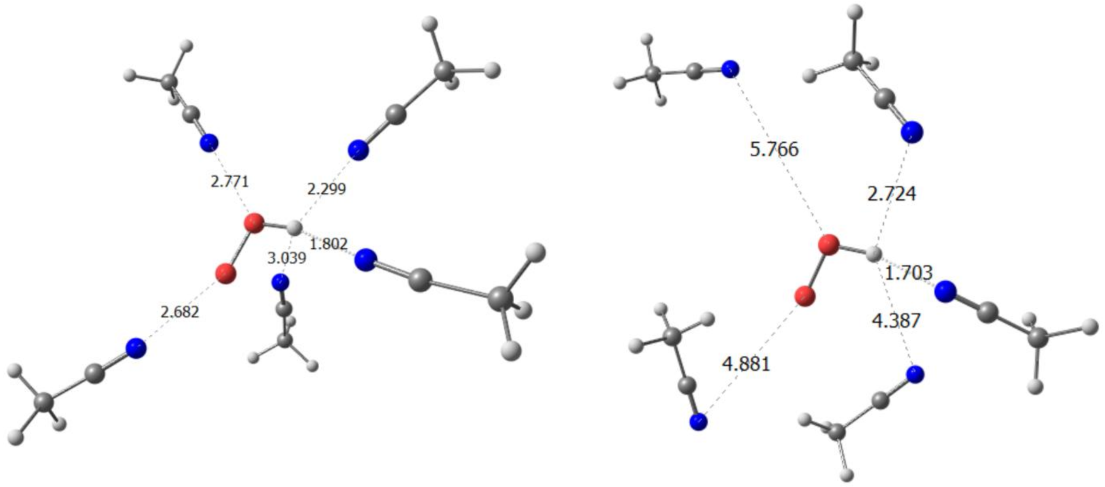

A: Starting structure for HOO-5ACN complex optimization.

B: Optimized structure for HOO-5ACN complex.

Figure S22. Optimization of the HOO$^{\bullet}$ radical with five acetonitrile molecules. (A) Starting geometry with solvent molecules positioned to interact with the oxygen lone pairs or the hydrogen-bond donor site. (B) Optimized structure, showing that only the acetonitrile molecule accepting the hydrogen bond remains close to the radical, while the others move away. These results indicate that binary $^{\bullet}$OOH---co-solvent complexes are the only relevant species for this study.

Table S2. Free energy data of the investigated species

CAM-B3LYP-GD3(BJ)/aug-cc-pVTZ						kbT/h	6.21E+12					
	G(1 atm)	G(1 M)										
	A.U.											
ACN.log	-132.724	-132.721										
MeOH.log	-115.699	-115.696										
DMSO.log	-553.185	-553.182										
Py.log	-248.19	-248.187										
DABCO.log	-345.139	-345.136										
CHD.log	-233.286	-233.283										
HOO.log	-150.94	-150.937										
F-	-100.003	-99.9995										
HMPA	-820.152	-820.149										
			ΔG(1 M) kcal/mol	Ks								
ACN-HOO.log	-283.664	-283.661	-2.09	34.1								
MeOH-HOO.log	-266.64	-266.637	-2.23	43.3								
DMSO-HOO.log	-704.132	-704.129	-5.87	2.0E+04								
Py-HOO.log	-399.139	-399.136	-7.19	1.9E+05								
DABCO-HOO.log	-496.09	-496.087	-9.05	4.3E+06								
			ΔG‡(1 M) kcal/mol	KtS				ΔG‡(kJ/mol)				
HOO-HOO-ACN.log	-434.592	-434.589	5.3	7.50E+08				22.3		9.00E+08		
HOO-HOO-MeOH.log	-417.567	-417.564	6.0	2.46E+08				25.1		7.00E+08		
HOO-HOO-DMSO.log	-855.06	-855.057	5.2	9.59E+08				21.7		4.00E+08		
HOO-HOO-Py.log	-550.066	-550.063	6.1	2.22E+08				25.4		1.00E+08		
HOO-HOO-DABCO.log	-647.022	-647.019	3.1	3.53E+10				12.8		6.00E+08		
				kpS	v(TS)	Q(Eckart)	kts*Q	ΔG‡(kJ/mol)		kts (expt)	k(calc)/k(expt)	
CHD-HOO-ACN.log	-516.917	-516.914	18.6	1.34E-01	-1876.94	467.4	62.82	77.9		130.0	0.48	
CHD-HOO-MeOH.log	-499.892	-499.889	19.2	5.24E-02	-1969.61	1057	55.36	80.3		90.0	0.62	
CHD-HOO-DMSO.log	-937.382	-937.379	20.5	5.85E-03	-1950.31	1147	6.71	85.7		20.0	0.34	
CHD-HOO-Py.log	-632.387	-632.384	21.9	5.61E-04	-2007.79	2377	1.33	91.5		5.0	0.27	
CHD-HOO-DABCO.log	-729.337	-729.334	22.6	1.61E-04	-2068.63	4627	0.75	94.6		0.1	7.45	

Table S3. Slope of rate of O_2 consumption during the autoxidation of CHD 0.26 M initiated by AIBN (25 mM) in MeCN at 30 °C, in the presence of increasing amounts of bases. Errors are within 20 %

Base concentration (mM)	slope $-d[O_2]/dt$ ($M\ s^{-1}$) **Pyridine**	slope $-d[O_2]/dt$ ($M\ s^{-1}$) **Piperidine**	slope $-d[O_2]/dt$ ($M\ s^{-1}$) **Me_4-piperidine**	slope $-d[O_2]/dt$ ($M\ s^{-1}$) **Tert-octylamine**	slope $-d[O_2]/dt$ ($M\ s^{-1}$) **Bu_4NOH**
0	1,34E-06	1,34E-06	1,34E-06	1,34E-06	1,34E-06
0.1	8,53E-07	1,07E-07	1,08E-07	2,48E-07	3,57E-08
1	8,28E-07	1,36E-08	4,20E-08	1,03E-07	
5	6,16E-07	1,69E-08	1,79E-08	7,02E-08	
30	2,00E-07	1,68E-08	2,82E-08	6,70E-08	

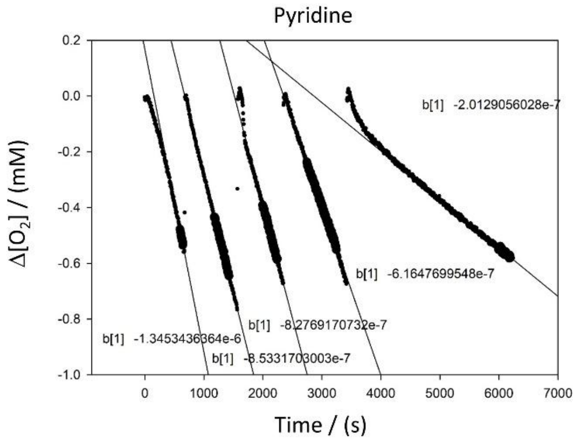

Figure S23. O_2 consumption during the autoxidation of CHD 0.26 M in ACN initiated by AIBN (25 mM) at 30 °C in the presence of increasing amounts of base.

Sample: 2 mL AIBN in ACN 0,05 M + 1,9 mL ACN + 100 μL CHD

+ 8 μL Py 50 mM in ACN (final concentration = 0.1 mM)

+ 70 μL Py 50 mM in ACN (final concentration = 1 mM)

+ 30 μL Py 0.5 M in ACN (final concentration = 5 mM)

+ 9 μL Py pure in ACN (final concentration = 30 mM)

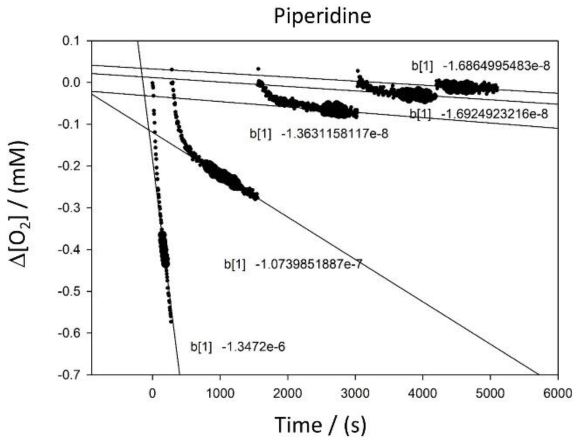

Figure S24. O_2 consumption during the autoxidation of CHD 0.26 M in ACN initiated by AIBN (25 mM) at 30 °C in the presence of increasing amounts of base.

Sample: 2 mL AIBN in ACN 0,05 M + 1,9 mL ACN + 100 μL CHD

+ 20 μL Piperidine 20 mM in ACN (final concentration = 0.1 mM)

+ 8 μL Piperidine 0.5 M in ACN (final concentration = 1 mM)

+ 20 μL Piperidine 0.8 M in ACN (final concentration = 5 mM)

+ 10 μL Piperidine pure in ACN (final concentration = 30 mM)

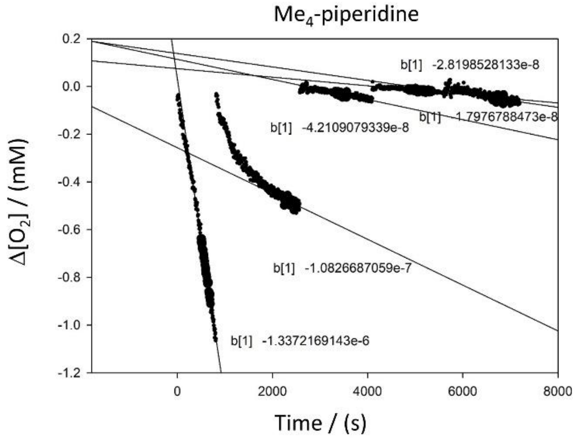

Figure S25. O_2 consumption during the autoxidation of CHD 0.26 M in ACN initiated by AIBN (25 mM) at 30 °C in the presence of increasing amounts of base.

Sample: 2 mL AIBN in ACN 0,05 M + 1,9 mL ACN + 100 μL CHD

+ 20 μL Me_4-piperidine 20 mM in ACN (final concentration = 0.1 mM)

+ 10 μL Me_4-piperidine 0.4 M in ACN (final concentration = 1 mM)

+ 20 μL Me_4-piperidine 0.8 M in ACN (final concentration = 5 mM)

+ 20 μL Me_4-piperidine pure in ACN (final concentration = 30 mM)

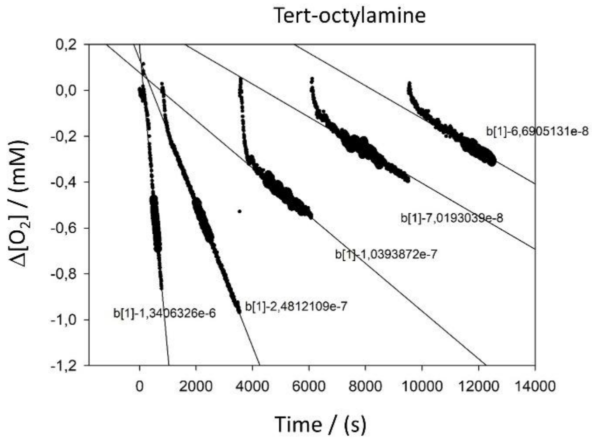

Figure S26. O_2 consumption during the autoxidation of CHD 0.26 M in ACN initiated by AIBN (25 mM) at 30 °C in the presence of increasing amounts of base.

Sample: 2 mL AIBN in ACN 0,05 M + 1,9 mL ACN + 100 μL CHD

+ 20 μL tert-octylamine 20 mM in ACN (final concentration = 0.1 mM)

+ 10 μL tert-octylamine 0.4 M in ACN (final concentration = 1 mM)

+ 20 μL tert-octylamine 0.83 M in ACN (final concentration = 5 mM)

+ 20 μL tert-octylamine pure in ACN (final concentration = 30 mM)

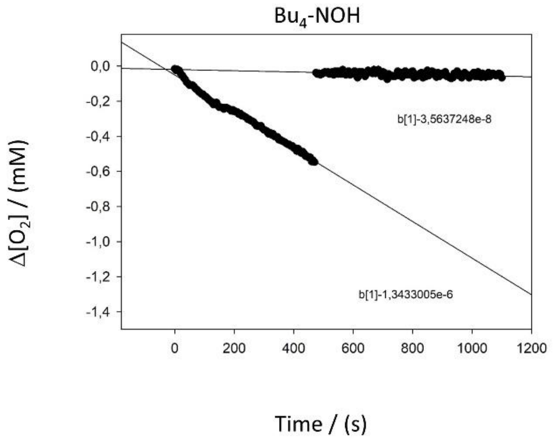

Figure S27. O_2 consumption during the autoxidation of CHD 0.26 M in ACN initiated by AIBN (25 mM) at 30 °C in the presence of increasing amounts of base.

Sample: 2 mL AIBN in ACN 0,05 M + 1,9 mL ACN + 100 μL CHD

+ 20 μL Bu_4NOH 20 mM in ACN (final concentration = 0.1 mM)

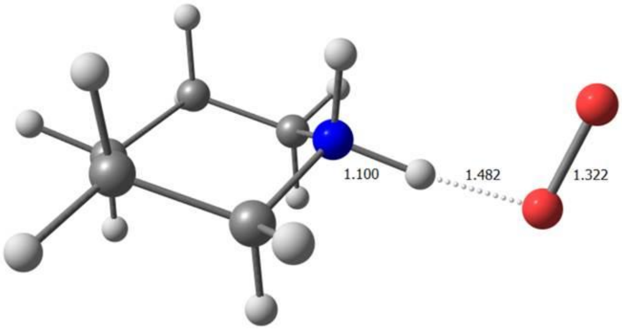

Figure S28. DFT optimizations of the piperidine-HOO• complex in acetonitrile implicit solvent.

Table S4. Optimized coordinates for the piperidine-HOO complex with acetonitrile implicit solvent (SMD).

6	-1.677644000	1.302151000	0.205652000
6	-0.248859000	1.189662000	-0.286623000
6	-0.383846000	-1.276991000	-0.200997000
6	-1.814312000	-1.199327000	0.292588000
6	-2.494299000	0.077126000	-0.187146000
1	-0.210082000	1.164914000	-1.375082000
1	0.368145000	2.014109000	0.061506000
1	-1.672106000	1.409268000	1.293258000
1	-2.116674000	2.211015000	-0.204335000
1	-0.347239000	-1.331830000	-1.288493000
1	0.140949000	-2.138101000	0.205087000
1	-2.351092000	-2.081250000	-0.055211000
1	-1.816299000	-1.230682000	1.385037000
1	-2.597507000	0.045013000	-1.274707000
1	-3.499148000	0.146539000	0.228198000
7	0.380649000	-0.066994000	0.188693000
1	0.460505000	-0.034833000	1.203311000
8	2.832412000	-0.181687000	-0.601890000
1	1.404554000	-0.136837000	-0.207837000
8	3.328239000	0.166176000	0.572836000

Reference

(1) Cardullo, N.; Monti, F.; Muccilli, V.; Amorati, R.; Baschieri, A. Reaction with ROO• and HOO• Radicals of Honokiol-Related Neolignan Antioxidants. *Molecules* **2023**, *28* (2), 735.

(2) Yanai, T.; Tew, D. P.; Handy, N. C. A new hybrid exchange–correlation functional using the Coulomb-attenuating method (CAM-B3LYP). *Chem. Phys. Lett.* **2004**, *393* (1-3), 51-57.

(3) Dunning, T. H. Gaussian basis sets for use in correlated molecular calculations. I. The atoms boron through neon and hydrogen. *J. Chem. Phys.* **1989**, *90* (2), 1007-1023.

(4) Kendall, R. A.; Dunning, T. H.; Harrison, R. J. Electron affinities of the first-row atoms revisited. Systematic basis sets and wave functions. *J. Chem. Phys.* **1992**, *96* (9), 6796-6806.

(5) Grimme, S.; Ehrlich, S.; Goerigk, L. Effect of the damping function in dispersion corrected density functional theory. *J. Comput. Chem.* **2011**, *32* (7), 1456-1465.

(6) Johnson, E. R.; Becke, A. D. A post-Hartree-Fock model of intermolecular interactions. *J. Chem. Phys.* **2005**, *123* (2), 24101.

(7) Marenich, A. V.; Cramer, C. J.; Truhlar, D. G. Universal solvation model based on solute electron density and on a continuum model of the solvent defined by the bulk dielectric constant and atomic surface tensions. *Journal of chemical physics B* **2009**, *113* (18), 6378-6396.

(8) M. J. Frisch, G. W. Trucks, H. B. Schlegel, G. E. Scuseria, M. A. Robb, J. R. Cheeseman, G. Scalmani, V. Barone, G. A. Petersson, H. Nakatsuji, X. Li, M. Caricato, A. V. Marenich, J. Bloino, B. G. Janesko, R. Gomperts, B. Mennucci, H. P. Hratchian, J. V. Ortiz, A. F. Izmaylov, J. L. Sonnenberg, Williams, F. Ding, F. Lipparini, F. Egidi, J. Goings, B. Peng, A. Petrone, T. Henderson, D. Ranasinghe, V. G. Zakrzewski, J. Gao, N. Rega, G. Zheng, W. Liang, M. Hada, M. Ehara, K. Toyota, R. Fukuda, J. Hasegawa, M. Ishida, T. Nakajima, Y. Honda, O. Kitao, H. Nakai, T. Vreven, K. Throssell, J. A. Montgomery Jr., J. E. Peralta, F. Ogliaro, M. J. Bearpark, J. J. Heyd, E. N. Brothers, K. N. Kudin, V. N. Staroverov, T. A. Keith, R. Kobayashi, J. Normand, K. Raghavachari, A. P. Rendell, J. C. Burant, S. S. Iyengar, J. Tomasi, M. Cossi, J. M. Millam, M. Klene, C. Adamo, R. Cammi, J. W. Ochterski, R. L. Martin, K. Morokuma, O. Farkas, J. B. Foresman, D. J. Fox,*Gaussian 16 Rev. C.01*; Wallingford, CT, 2016.
